# Improving hematopoietic recovery through modeling and modulation of the mesenchymal stromal cell secretome

**DOI:** 10.1186/s13287-018-0982-2

**Published:** 2018-10-24

**Authors:** Frances D. Liu, Kimberley Tam, Novalia Pishesha, Zhiyong Poon, Krystyn J. Van Vliet

**Affiliations:** 1 Department of Biological Engineering, Massachusetts Institute of Technology, 77 Massachusetts Avenue, Cambridge, MA 02139 USA; 2 Biosystems and Micromechanics (BioSyM) Interdisciplinary Research Group, Singapore-MIT Alliance for Research and Technology, 1 Create Way, Singapore, 138602 Singapore; 3 Whitehead Institute for Biomedical Research, 455 Main Street, Cambridge, MA 02139 USA; 4 Department of Materials Science and Engineering, Massachusetts Institute of Technology, 77 Massachusetts Avenue, Cambridge, MA 02139 USA

**Keywords:** Mesenchymal stromal cells, Secretome, Mechanobiology, Hematopoietic recovery, Radiation injury

## Abstract

**Background:**

Efficient and sustained hematopoietic recovery after hematopoietic stem cell or bone marrow transplantation is supported by paracrine signaling from specific subpopulations of mesenchymal stromal cells (MSCs). Here, we considered whether in vitro mechanopriming of human MSCs could be administered to predictively and significantly improve in vivo hematopoietic recovery after irradiation injury.

**Methods:**

First, we implemented regression modeling to identify eight MSC-secreted proteins that correlated strongly with improved rescue from radiation damage, including hematopoietic recovery, in a murine model of hematopoietic failure. Using these partial least squares regression (PLSR) model parameters, we then predicted recovery potential of MSC populations that were culture expanded on substrata of varying mechanical stiffness. Lastly, we experimentally validated these predictions using an in vitro co-culture model of hematopoiesis and using new in vivo experiments for the same irradiation injury model used to generate survival predictions.

**Results:**

MSCs grown on the least stiff (elastic moduli ~ 1 kPa) of these polydimethylsiloxane (PDMS) substrata secreted high concentrations of key proteins identified in regression modeling, at concentrations comparable to those secreted by minor subpopulations of MSCs shown previously to be effective in supporting such radiation rescue*.* We confirmed that these MSCs expanded on PDMS could promote hematopoiesis in an in vitro co-culture model with hematopoietic stem and progenitor cells (HSPCs). Further, MSCs cultured on PDMS of highest stiffness (elastic moduli ~ 100 kPa) promoted expression of CD123^+^ HSPCs, indicative of myeloid differentiation. Systemic administration of mechanoprimed MSCs resulted in improved mouse survival and weight recovery after bone marrow ablation, as compared with both standard MSC expansion on stiffer materials and with biophysically sorted MSC subpopulations. Additionally, we observed recovery of white blood cells, platelets, and red blood cells, indicative of complete recovery of all hematopoietic lineages.

**Conclusions:**

These results demonstrate that computational techniques to identify MSC biomarkers can be leveraged to predict and engineer therapeutically effective MSC phenotypes defined by mechanoprimed secreted factors, for translational applications including hematopoietic recovery.

**Electronic supplementary material:**

The online version of this article (10.1186/s13287-018-0982-2) contains supplementary material, which is available to authorized users.

## Background

Hematopoietic stem cell (HSC) transplantations are performed as curative treatments for hematological malignancies such as leukemias, lymphomas, and myelomas [1, 2]. Successful HSC transplantation with rapid hematopoietic recovery and long-term HSC engraftment remains a challenge. HSCs can fail to engraft or can proliferate and differentiate too slowly, resulting in immune deficiency and infection susceptibility during post-transplantation recovery [3, 4]. Of equal concern, upon immune system suppression, the administered cells can overtake the recipient’s cells, resulting in graft-versus-host disease [1, 2, 5–7]. These complications related to the HSC transplantations, but non-cancer related transplantations have a reported mortality as high as 40–50% [1, 2]. Myeloablative conditioning regimens, such as radiation or chemotherapy, are used commonly to reduce tumor burden and to suppress immune rejection of the transplant [8–10]. Many of these regimens destroy both diseased and healthy cells of the hematopoietic compartment, including the stroma and niche-associated cell types. Subsequent HSC transplantation is necessary to replace the destroyed hematopoietic lineages, but HSCs do not repair the stromal or niche cells [11]. HSC engraftment and rapid, successful hematopoietic recovery are dependent on the hematopoietic niche components, including paracrine signaling and interactions with surrounding cell types [12–16].

Mesenchymal stromal cells (MSCs) are a subset of hematopoietic niche cells important in supporting, maintaining, and expanding hematopoietic stem and progenitor cells (HSPCs) in vivo and in vitro [13, 17–20]. MSC support of hematopoiesis in vivo and in vitro is attributed to MSC secretion of soluble factors such as stromal cell-derived factor 1 (SDF1) and stem cell factor (SCF) [21–25]. These cells natively reside in the bone marrow niche and also contain a subset of multipotent stem cells that can differentiate into osteogenic, chondrogenic, and adipogenic lineages [26, 27]. Due in part to this capacity for multipotent differentiation potential and asymmetric division, the MSC population becomes morphologically and functionally heterogeneous under standard ex vivo expansion conditions [28, 29]. We demonstrated previously that culture-expanded MSCs, which are otherwise immunophenotypically indistinguishable can be isolated into biophysically distinct subpopulations that result in distinct functional phenotypes [30, 31]. We found that the biophysical marker of suspended cell diameter correlated with varying ability of the MSCs to support hematopoietic recovery in vivo [30]. In that study, we systemically administered MSC populations or distinct MSC subpopulations (isolated by cell diameter via spiral microfluidics) into sub-lethally irradiated mouse models of hematopoietic failure analogous to irradiation used for myeloablative conditioning regimens [30]. We found that MSCs of larger cell diameter (*D*^hi^ MSCs) improved radiation rescue over any other isolated subpopulation or the heterogeneous MSC populations [30]. These *D*^hi^ MSCs had limited differentiation potential characteristic of osteoprogenitors [30, 31], which have also been demonstrated to support hematopoietic recovery in vivo [32–34]*.* In that prior in vivo study, the *D*^hi^ MSCs or osteoprogenitors did not exhibit long-term engraftment (> 10 days), and mouse survival also improved (though less dramatically) with injection of conditioned media from the *D*^hi^ MSCs. Those findings suggesting that the MSCs were capable of indirectly supporting hematopoietic recovery via paracrine signaling [30], as MSCs are not known to differentiate into hematopoietic lineages in vivo.

While such label-free biophysical sorting of MSCs is an effective approach to identify and isolate MSC subpopulations of therapeutic value, the *D*^hi^ MSCs were only a minor fraction of the culture-expanded population (20–30%). Correspondingly, the production yield of this osteoprogenitor phenotype was low. We have also demonstrated recently that the MSC secretome can be modulated by in vitro expansion on a viscoelastic polymer of tunable mechanical stiffness, polydimethylsiloxane (PDMS) [35]. By expanding MSCs on PDMS of decreasing stiffness, we increased the expression of various growth factors and cytokines such as osteopontin (OPN), interleukin-8 (IL-8), insulin-like growth factor binding protein 2 (IGFBP2), monocyte chemoattractant protein-1 (MCP-1), and SDF1α [35]. These secreted factors have been shown in the literature to maintain HSC self-renewal capacity in vivo*,* regulate HSC differentiation in vivo, or support ex vivo expansion of long-term re-populating HSCs [21, 36–44]. Many of these secreted factors were also overexpressed in biophysically sorted *D*^hi^ MSCs. Thus, we hypothesized that by growing the MSCs on PDMS substrata of designed mechanical stiffness, we could predictively produce unsorted, mechanoprimed MSC populations that would support hematopoietic recovery in vivo to levels comparable to that achieved by administering the minor subpopulation of *D*^hi^ MSCs.

In the current study, we constructed a regression model from the secretome expression of the MSC populations and subpopulations that support recovery after bone marrow irradiation (i.e., radiation rescue) including hematopoietic recovery to varying extents in vivo [30]. Those expression data served as training set to predict survival after hematopoietic failure, in response to administration of different MSC preparations. We then used the expression profile of the MSCs expanded on PDMS, or mechanoprimed MSCs, as test data to validate the regression model. We verified that our mechanoprimed MSCs could promote radiation rescue using this regression model, and potentially could modulate the differentiation and proliferation of hematopoietic stem and progenitor cells (HSPCs) under in vitro co-culture. Lastly, we then deployed our mechanoprimed MSCs in the same in vivo model to demonstrate improved hematopoietic recovery in similar ways predicted by the statistical regression model.

## Methods

### Regression modeling

We compiled all MSC secretome expression data that were acquired from previous studies using Luminex-based assays or enzyme-linked immunosorbent assays (ELISAs) [30]. Expression data and survival curves were collected from five experimental groups: unsorted MSCs at passages 3, 6, and 9 (approximate population doublings of 6, 12, and 18) expanded on tissue culture polystyrene (TCPS); and two subpopulations of size-sorted MSCs (*D*^hi^ and *D*^lo^ MSCs) expanded to passage 6 on TCPS. Survival curves were acquired over 21 time points during the 50-day experiment duration. We first calculated the Pearson’s linear correlation coefficients among the expression data of 35 secreted factors against the survival proportions at a single time point (e.g., survival proportions for each experimental group at day 18). We then applied this same analysis to every time point to determine all proteins or cytokines that were significantly (*p* < 0.05), linearly correlated (|ρ| > 0.875) with survival over the duration of the entire experiment. As there is no *a priori* expectation that the relationship between cytokine expression and survival is linear, we also conducted partial least squares regression (PLSR) to determine what proteins and cytokines were most strongly correlated with survival. For PLSR, the expression data were input as a 5 × 35 matrix of predictors while the survival curve data were input as a 5 × 21 response matrix. For both predictor and response matrices, we z-score normalized each column to have a mean of 0 and standard deviation of 1; this approach obviated inappropriate weighting of variables based on relative magnitude (i.e., concentration). Over 90% variance in both the predictor and response matrices was contained within a two-component model; thus, we chose to use two-dimensional principal component space to project our loading vectors (see Additional file 1: Figure S1). We determined which secreted factors correlated most strongly with survival by determining the loading vectors of the predictor and response matrices that were closest together. Using this PLSR model, we also obtained a 36 × 21 matrix of regression coefficients, with the top row as intercepts, which could be used to predict survival using new expression data of the 35 proteins and secreted factor included in the analysis. We conducted all computations in MATLAB and the Statistics and Machine Learning Toolbox.

### MSC culture

We prepared PDMS-based cell culture substrata with tunable viscoelastic properties as described previously [35]. Briefly, we mixed a two-component PDMS (CY 52–276, Dow Corning, Midland, MI, USA) at three different mass ratios to form substrata of shear elastic moduli varying over three orders of magnitude (~ 1 kPa, ~ 10 kPa, ~ 100 kPa). We then added the PDMS mixtures to polystyrene well-plates or petri dishes at volumes sufficient to form PDMS layers of ~ 500 μm thickness and cured these at 80 °C for ~ 24 h. We plasma-treated PDMS surfaces for 5 min to render them sufficiently hydrophilic for cell attachment. We then cultured human bone marrow-derived MSCs on these PDMS substrata as described previously [35]. Prior to using the MSCs for these experiments, the MSCs were commercially purchased (Lonza, Basel, Switzerland) and expanded on tissue culture polystyrene up to passage 5–7. All expansion media (for both HSPCs and MSCs) and growth conditions were prepared as described previously [35].

For secretome characterization and co-culture with HSPCs, we cultured MSCs on plasma-treated PDMS in 12-well plates. For both of these in vitro experiments, we plated MSCs at high densities (~ 10,000 cells/cm^2^) to ensure MSC confluency and growth-arrest within 4–5 days to maintain approximately constant cell numbers across all experimental conditions. For secretome characterization in 12-well plates, smaller volumes of media (1 mL/well) were then added to the wells upon media exchange at day 3 or 4. We then allowed the MSCs to condition the smaller volume of fresh MSC expansion media for 4–5 days prior to harvesting the media from the wells for secretome characterization. To account for potential differences in MSC number across conditions, we fixed and stained the cells for nuclei (Hoechst 33342) after harvesting the secretome samples. We imaged at least 10 locations in each well to verify that MSC numbers were approximately constant across all conditions. If they were not, we normalized the concentrations by cell number normalization factors as described previously [35].

For sufficient expansion of MSCs for mouse studies, we cultured the MSCs on dishes of larger growth area: 150 mm-diameter petri dishes (Thermo Fisher Scientific, Waltham, MA, USA; Cat. No. 157150). We plated MSCs at a seeding density of ~ 1500–2000 cells/cm^2^ and allowed the MSCs to proliferate for 7–10 days with MSC expansion media replaced every 3–4 days. We expanded MSCs on two PDMS substrata of lowest and highest stiffness: 1 kPa and 100 kPa. As a comparison to our previous studies, we included additional experimental groups: MSCs expanded on tissue culture polystyrene dishes (termed unsorted MSCs) and subsequently sorted biophysically (termed *D*^hi^ MSCs) as described previously [30].

### Secretome characterization

For secretome characterization, we assayed five MSC conditions: *D*^hi^ MSCs and unsorted MSCs expanded on tissue culture polystyrene as well as unsorted MSCs that were culture-expanded on PDMS of 1, 10, or 100 kPa stiffness. After allowing the MSCs to condition the smaller volumes of expansion media for 4–5 days, we harvested the MSC-conditioned media for secretome characterization. After harvesting secretome samples, we centrifuged them for 8 min at 500 g to remove any cells or cell debris. We then transferred the supernatant to new tubes, frozen down at − 80 °C prior to use.

We thawed the samples on ice prior to protein characterization and assayed composition in technical duplicates using the ProcartaPlex 45-plex human cytokine/chemokine/growth factor panel (Thermo Fisher Scientific, EPX450–12171-901) following the manufacturer’s instructions. We conducted washing steps using a microplate washer (BioTek, Winooski, VT, USA) and subsequently read the concentrations with the FlexMap 3D (Luminex, Austin, TX, USA). To assay the secretome samples for concentrations of angiopoietin-1 (ANG-1), bone morphogenetic protein 2 (BMP-2), acidic fibroblast growth factor (FGF-1), and thrombopoietin (THPO), which were not included in the Luminex-based panel, we completed multiple ELISAs (R&D Systems, Minneapolis, MN, USA; Cat # DANG10, DBP200, DFA00B, and DTP00B). Concentrations of BMP-2 and THPO in our samples were below the detection limit. We used the arithmetic mean as representative concentrations for all experimental groups for PLSR model predictions.

### MSC and HSPC co-culture

We refer to mobilized human bone marrow-derived CD34^+^ cells (from the Fred Hutchinson Cancer Research Center, Seattle, WA, USA) as hematopoietic stem and progenitor cells (HSPCs). After the same 4–5 days of MSC expansion on top of PDMS wells as conducted for secretome characterization, we aspirated to remove the MSC expansion media and directly added HSPCs on top of the MSCs. In the co-culture experiments, we co-cultured HSPCs with unsorted MSCs grown on the three PDMS substrata and on tissue culture polystyrene as a comparison to standard culture practice. We expanded the HSPCs in expansion media on top of MSCs for ~ 5–8 days as described previously [35]. We aspirated to harvest HSPCs from the MSCs and collected any remaining HSPCs with vigorous PBS washing and collection. To determine proliferation, we counted HSPCs with the Cellometer Auto T4 Cell Viability Counter (Nexcelom Bioscience, Lawrence, MA, USA) for all conditions. We conducted flow cytometry as described previously to assay HSPC surface marker expression of CD34 (eBioscience, San Diego, CA, USA) 11–0349-42) and CD123 (eBioscience 48–1238-42) [35].

### In vivo model

Procedures involving animals and their care were conducted in conformity with all procedures approved by the National University of Singapore Institutional Animal Care and Use Committee (IACUC), and all in vivo studies reported herein occurred in Singapore at the National University of Singapore’s animal housing facility. We compared in vivo responses for four distinct preparations of MSCs: unsorted MSCs expanded on tissue culture polystyrene; *D*^hi^ MSCs sorted from MSCs expanded on tissue culture polystyrene, and unsorted MSCs grown on 1 kPa PDMS or 100 kPa PDMS. All MSCs used for injection were derived from the same human donor (purchased from Lonza) and expanded to the same passage number under identical culture conditions as described above.

We purchased and used 5–7 week old, female immune-compromised mice (NOD SCID, The Jackson Laboratory, Bar Harbor, ME, USA) in irradiation studies as described previously [30]. We irradiated the mice using 4.0 Gy of gamma irradiation to induce hematopoietic failure. At 24 h post-irradiation, we injected MSCs (trypsinized and re-suspended at 10^6^ MSCs/mL) via tail vein injection at a concentration of ~ 20,000 MSCs/g of mouse mass. As a negative vehicle control (no-treatment), we included a cohort injected with only saline, for a total of five condition cohorts. We tracked mouse survival and weight loss over the 50-day duration of the experiment. At weeks 1, 2, and 4 post-irradiation, we collected blood via cardiac puncture for complete blood counts (CBC), culling three randomly selected mice from the experimental groups injected with MSCs that were expanded on PDMS (1 kPa and 10 kPa). For the first two time points, we also culled and collected blood from mice in the no-treatment, saline control cohort. At week 5 (day 35), we also collected a small volume of blood (~ 0.1–0.3 mL) from at least five mice in the two experimental groups for CBC using a facial sub-mandibular bleed. We analyzed all in vivo data and corresponding statistics using GraphPad Prism 7 (GraphPad Software, San Diego, CA, USA).

## Results

### Regression modeling and training data

**Fig. 1:**
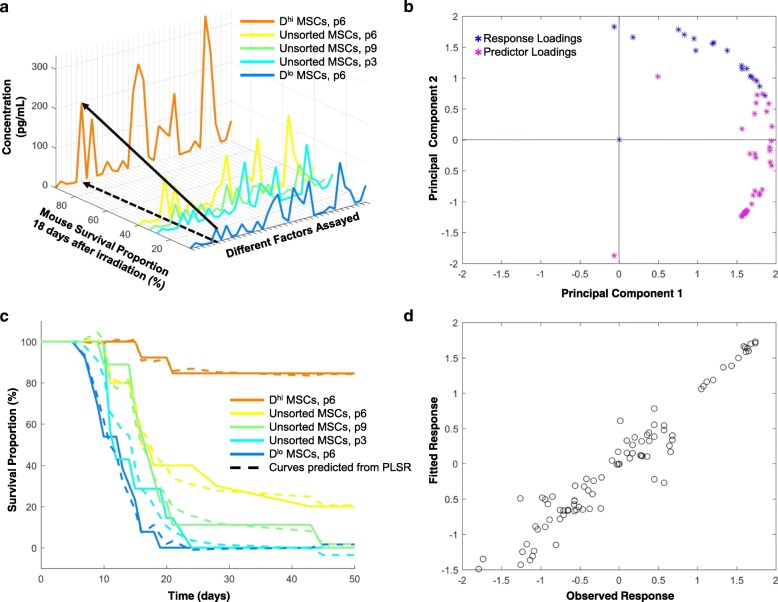
Training data and regression modeling. **a** Expression data of 35 secreted proteins versus the mouse survival proportions at 18 days post-irradiation. Secreted protein concentrations were assayed prior to injection for five experimental MSC groups: *D*^hi^ MSCs (*orange*), unsorted MSCs at three different passages (*yellow, green, cyan*), and *D*^lo^ MSCs (*blu*e). The small “p” indicates passage number of the cell population, e.g., p6 is passage 6 or approximately 12 population doublings in vitro. *Black arrows* visualize a single protein’s increasing concentration trending with increasing survival proportion. **b** Loading vectors of predictor (*purple stars*) and response (*blue stars*) variables in two-dimensional principal component plot from PLSR. **c** Survival curves from all five experimental groups are plotted with survival curves predicted from PLSR model (*dashed line*). **d** Every data point from the model rendered as fitted data against the experimental or observed data

The first goal of the present study was to identify any key factors, cytokines, or proteins that correlated or anti-correlated with improved survival indicative of hematopoietic recovery. We had characterized previously the secretome expression of five distinct MSC populations using targeted, antibody-based assays including a Luminex-based panel and multiple ELISAs [30]. We had also tracked the survival of sub-lethally irradiated mice injected with those same MSC populations, as a model of hematopoietic failure and recovery [30]. Here, we related those previously acquired secretome expression data against the survival proportions of the mice at a given time point (i.e, at day 18, Fig. 1a). From this three-dimensional graph, we visualized linear trends and calculated linear correlation coefficients for which increased survival proportion increased with expression of a specific protein (indicated by black arrows, Fig. 1a). However, the relative survival proportions of the mice across all five experimental groups varied throughout the 50-day duration of the experiment. Thus, we generated a similar three-dimensional plot for all 21 time points of the experiment. For each time point, we then calculated the linear Pearson correlation coefficient. Table 1 (column 1) indicates the cytokines and growth actors with significant (*p* < 0.05) linear Pearson correlation coefficients (|ρ| > 0.875). We considered several different passages of the MSCs, given our group’s previous and ongoing work regarding the emergent heterogeneity of MSCs under typical in vitro culture expansion pressures; p6 represents passage 6 or approximately 12 population doublings in vitro under the expansion culture conditions at the time of that study used to acquire the mouse survival data, and as described previously [30].

**Table 1:** Proteins expressed by MSCs and significantly correlated with mouse survival, post-irradiation

Pearson linear regression	Partial least squares regression	Overlapping proteins
ANG-1	IL-6	IL-6
BMP2	IL-8	IL-8
EGF	BMP2	BMP2
FGF1	EGF	EGF
IL-15	FGF1	FGF1
IL-6	MCP-1	RANTES
IL-8	RANTES	VEGF-A
RANTES	VEGF-A	ANG-1
THPO	ANG-1	
VEGF-A		

Overlapping proteins indicate those proteins identified as correlated significantly by both Pearson linear regression and partial least squares regression

*ANG-1* angiopoietin-1, *BMP-2* bone morphogenetic protein 2, *EGF* epidermal growth factor, *FGF-1* fibroblast growth factor 1*, IL-6* interleukin 6, *IL-8* interleukin 8, *IL-15* interleukin 15, *MCP-1* monocyte chemoattractant protein 1, *RANTES* regulated upon activation, normally T-expressed, and presumably secreted, also known as CC-motif chemokine ligand 5, *THPO* thrombopoieitin, *VEGF-A* vascular endothelial growth factor A.

As a linear correlation between MSC secreted factors and mouse survival is not anticipated *a priori*, we also conducted partial least squares regression (PLSR) as a separate statistical regression method to determine secreted factors significantly correlated with survival. A two-component model was sufficient to capture over 90% of the variance in both the response (survival) and predictor (expression) variables (see Additional file 1: Figure S1). Figure 1b shows the predictor and response loading vectors projected into the two-dimensional principal component space calculated from PLSR. Each predictor loading vector represents the survival proportions at each time point of the study, while each response loading vector includes the expression of each of the 35 proteins detected and analyzed in the secretome. The predictor loading vectors closest to the response loading vectors were those growth factors and cytokines that were correlated most strongly with survival. In this model, we did not observe any secreted factors that were anti-correlated with survival. Table 1 (column 2) indicates the proteins most highly correlated with increased survival as determined from PLSR. We calculated the regression coefficients and intercepts from PLSR to construct predicted survival curves. Using the same model data, we used PLSR to predict survival curves for the five groups considered in those previously reported in vivo experiments (Fig. 1c-d). The predicted survival curves (*dotted* lines, Fig. 1c) corresponded closely with the original model data (solid lines, Fig. 1c). Figure 1d also indicates parity between our regression model and those experimental data, with a slope of approximately unity when graphing the predicted or fitted response from PLSR against the original training data.

Table 1 also indicates the cytokines and proteins that were identified from both statistical regression methods, suggesting strong correlations with improved survival. These secreted factors included interleukin-6 (IL-6), interleukin-8 (IL-8), bone morphogenetic protein 2 (BMP2), epidermal growth factor (EGF), fibroblast growth factor 1 (FGF1), regulated on activation, normal T cell expressed and secreted (RANTES), vascular endothelial growth factor A (VEGF-A), and angiopoietin-1 (ANG-1). We calculated significant Pearson correlations for Thrombopoietin (THPO) and interleukin-15 (IL-15), but these factors were not also identified in PLSR. On the other hand, monocyte chemoattractant protein 1 (MCP-1) was identified in PLSR, but not in linear Pearson correlations. Additional file 1: Figure S2 shows the same three-dimensional rendering (Fig. 1a) with all factor names designated and highlights those factors identified from both methods noted in Table 1.

### Secretome modulation and training data

**Fig. 2:**
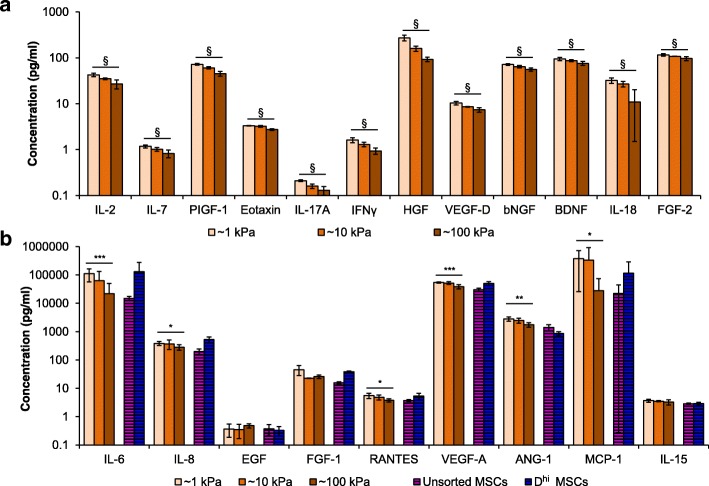
Secretome characterization and test data. **a** Unsorted MSCs were expanded on all PDMS substrata of varying stiffness (1, 10, and 100 kPa PDMS) for 7 days, represented *by light, medium, and dark orange colors*, respectively.) Expression of MSC secreted proteins that have significant trends (§*p* < 0.001) with cell culture substratum stiffness as determined from Spearman’s rank-based correlation. **b** As a comparison to MSCs on PDMS, unsorted and *D*^hi^ MSCs were also grown on TCPS and are represented by *striped purple and blue bars*, respectively. Expression of MSC secreted proteins identified in Table 1 were assayed across all five different culture conditions. Expression of MSC secreted proteins that have significant trends (*p < 0.05, **p < 0.01, ***p < 0.005) with cell culture substratum stiffness as determined from Spearman’s rank-based correlation. All data are plotted as arithmetic mean ± standard deviation

To obtain new training data for this model correlating the mechanically modulated MSC secretome with mouse survival, we expanded MSCs on PDMS substrata of stiffness varied across three orders of magnitude (shear elastic modulus *G* of ~ 1, 10, or 100 kPa, as characterized previously [35]). We then characterized the resulting conditioned media using a Luminex-based panel and multiple ELISAs. We calculated the Spearman rank-based correlation between substratum stiffness and expression, and identified significant correlations (*p* < 0.001) for the concentrations of a dozen different secreted proteins (Fig. 2a). Because we calculated correlations for every factor assayed, we corrected the critical *p*-value using a Bonferroni correction to account for multiple hypothesis testing. This analysis indicated that the concentrations of these dozen factors increased with decreasing substratum stiffness.

In addition to characterizing the MSCs grown on PDMS, we also characterized the secretome of MSCs grown on polystyrene in parallel, as a standard of comparison to our previous results and as a typical culture condition for MSCs used in preclinical studies. Specifically, we characterized the secretome of unsorted MSCs and *D*^hi^ MSCs from the same MSC donor grown on tissue culture polystyrene (TCPS). In all subsequent results, we indicated data for MSCs expanded on TCPS as black-striped bars, to emphasize that these MSCs were grown on a material with surface chemistry and topography distinct from PDMS (Fig. 2b). Of course, TCPS is also significantly stiffer (~ 1 GPa) than the PDMS substrata that we considered herein (1–100 kPa), but we do not draw inferences of mechanical cues from comparisons with TCPS due to the other marked differences between these polymeric surfaces.

Figure 2b shows the concentrations of nine secretome factors identified through statistical analysis to be correlated with survival, for five experimental groups of MSC expansion conditions (Table 1). Similar to the expression data obtained in previous experiments [30], the *D*^hi^ cells exhibited higher expression of these factors than the unsorted MSCs (Fig. 2b). For the factors statistically correlated with improved hematopoietic recovery, we observed that MSCs expanded on the most compliant PDMS substrata (1 kPa) exhibited expression levels similar to or greater than that of the *D*^hi^ MSC subpopulations (Fig. 2b). We used these secretome data for subsequent model predictions.

### In vitro validation

**Fig. 3:**
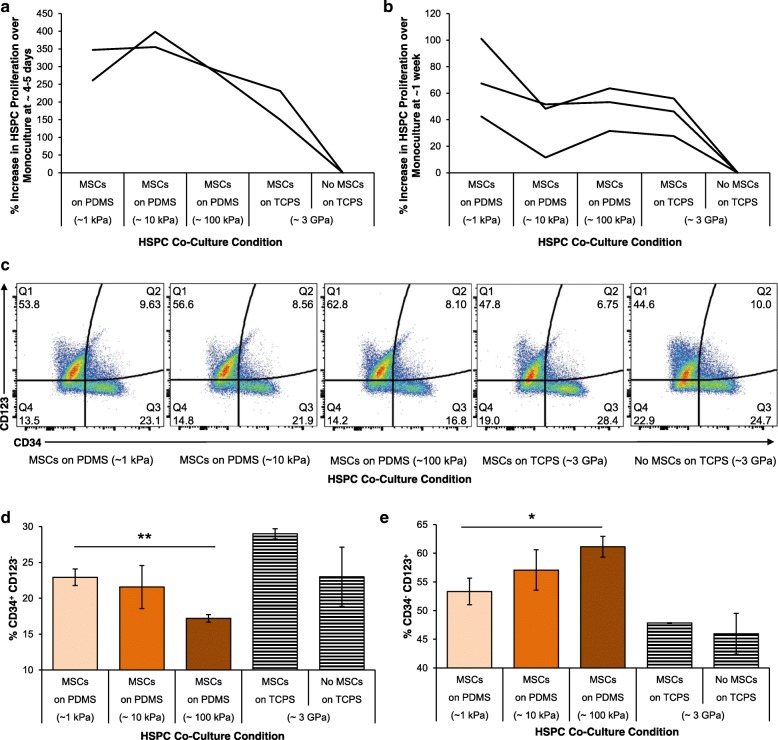
In vitro hematopoietic recovery: HSPC and MSC co-culture. Hematopoietic stem and progenitors (HSPCs) were grown in direct contact co-culture with MSCs grown on 1, 10, 100 kPa PDMS and TCPS. Proliferation of HSPCs in these co-culture conditions relative to monoculture were determined at (**a**) 4–5 days and (**b**) 1 week after co-culture. Individual lines represent replicate experiments. **c** HSPC surface marker expression of CD123 and CD34 assayed using flow cytometry. **d** % expression of CD34^+^ and CD123^−^ cells as mean ± standard deviations. **e** % expression of CD34^−^ and CD123^+^ cells as mean ± standard deviations. **d**-**e** Co-culture on PDMS of increasing stiffness (1, 10, 100 kPa) indicated in *darkening shades of orange*, respectively. Co-culture and monoculture of HSPCs on tissue culture polystyrene are shown as with *black- and white-striped bars* as distinct current standard protocols for comparison. Significant differences were calculated with one-tailed Student’s *t* tests with unequal variance, (*p < 0.01, **p < 0.005). Data are plotted as mean ± standard deviation, *N* = 3 across replicate wells

To determine whether these mechanoprimed MSCs could plausibly promote hematopoiesis in vivo, we first considered an in vitro assay for hematopoietic stem/progenitor cell proliferation. Specifically, we co-cultured our mechanoprimed MSCs with human hematopoietic stem and progenitor cells (HSPCs, CD34+). These HSPCs are the population of stem and progenitors that repopulate the blood cell lineages of the bone marrow. As a control and standard of comparison, we also expanded the HSPCs in monoculture. We found that HSPC proliferation was increased when grown in co-culture with MSCs that were adhered to the most compliant PDMS substrata, both at day 4–5 in co-culture (Fig. 3a) and after 1 week in co-culture (Fig. 3b). We also characterized the surface marker expression of the HSPCs after co-culture via flow cytometry. Figure 3c shows representative dot plots, wherein quadrant 3 (the CD34^+^CD123^−^ population) demonstrated a significant increase (**p < 0.005) in CD34^+^ expression for HSPCs co-cultured on the more compliant of the two PDMS substrata (Fig. 3d). When considering the common myeloid progenitor marker CD123, also known as IL-3Rα, we found that the CD123^+^CD34^−^ expression was increased for co-culture with MSCs grown on either of those PDMS substrata as compared with the current standard, TCPS. Moreover, we found that this myeloid priming was increased significantly (*p < 0.01) when HSPCs were grown in co-culture with MSCs on 100 kPa PDMS (Fig. 3e).

### In vivo validation

**Fig. 4:**
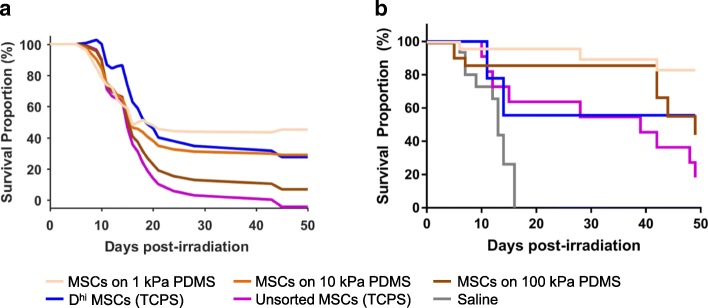
In vivo hematopoietic recovery: mouse survival. **a** Survival curves were predicted using the PLSR model for mice injected with unsorted MSCs grown on PDMS (*three orange lines*), *D*^hi^ MSCs (*blue line*) on TCPS, and unsorted MSCs (*purple line*) on TCPS. PDMS substrata of varying stiffness (1, 10 and 100 kPa PDMS) represented by *light, medium, and dark orange colors*, respectively. **b** Mice were irradiated with 4.0 Gy gamma irradiation at day 0 and injected with MSCs from four experimental groups at day 1. These four experimental groups included unsorted MSCs grown on 1 kPa PDMS (*light orange*), 100 kPa (*dark orange*) PDMS, TCPS (*purple)* and *D*^hi^ MSCs grown on TCPS (*blue*). A no-treatment or saline control is also included in *gray*. Mice survival for each group was tracked over the course of 50 days after irradiation and *N* ≥ 9 for all conditions. For cohorts injected with saline, unsorted MSCs on TCPS, and ~ 100 kPa PDMS, median survival times were 13, 39, and 49 days, respectively. Median survival times were undefined (> 50 days) for *D*^hi^ MSCs and unsorted MSCs on ~ 1 kPa PDMS

After observing increased HSPC proliferation and changes in HSPC surface marker expression in vitro (Fig. 3) when grown in co-culture with our mechanoprimed MSCs, we next considered how these mechanoprimed MSCs could affect hematopoietic recovery in vivo. Using our regression model (Fig. 1) and the characterized secretome expression of the mechanoprimed MSCs (Fig. 2), we first predicted the survival graphs using our PLSR model and measured expression data (Fig. 4a). Injection of MSCs expanded on 1 kPa PDMS was predicted to exhibit the highest endpoint survival of ~ 50% at day 50, the duration of the experiment (Fig. 4a). When considering only the MSCs expanded on TCPS (blue and purple lines), as expected, the *D*^hi^ cells were predicted to elicit a greater survival percentage than achieved with the unsorted cells over the entire experiment duration (Fig. 4a). Among the PDMS substrata (three orange lines), mice were predicted to exhibit higher survival percentages when injected with MSCs grown on PDMS substrata of decreasing stiffness (Fig. 4a). In other words, mouse survival was predicted to be maximized for unsorted MSCs grown on PDMS of stiffness 1 kPa.

We observed that several of these predicted relative trends were replicated accurately in our animal model experiment (Fig. 4b). For example, the administration of MSCs grown on 1 kPa PDMS substrata yielded the highest survival with significantly higher survival curves than MSCs grown on 100 kPa PDMS and unsorted MSCs grown on TCPS (*p* = 0.0027 and 0.0001, respectively, as calculated from Mantel-Cox or log-rank test). Also consistent with the PLSR predictions, the endpoint survival proportion of mice injected with MSCs grown on 1 kPa PDMS was greater than for *D*^hi^ MSCs (83.2% vs. 55.6%); this survival percentage for MSCs on 1 kPa PDMS exceeded our PLSR model prediction. However, the difference between these mechanoprimed and sorted *D*^hi^ MSCs was not statistically significant (*p* > 0.05). Additionally, the mouse cohort injected with MSCs grown on 100 kPa declined below that of the 1 kPa cohort at ~ 40 days post-irradiation. Finally, in the cohort injected with the *D*^hi^ cells, we observed a near 50% drop in survival within the first 15 days, which then plateaued (Fig. 4b).

Certain findings were not predicted precisely by the PLSR model. For example, for the first 40 days of the study, unsorted MSCs cultured on both PDMS substrata (100 kPa and 1 kPa) yielded higher survival proportions than the *D*^hi^ cells. The PLSR model predicted cohorts administered with the unsorted MSCs on TCPS to fare worse (i.e., lower survival percentages by day 40) than observed. In fact, the mouse cohort injected with unsorted MSCs on TCPS fared similarly to the *D*^hi^ cells up to day 37 post-treatment, and only later proceeded to decline rapidly. The model also predicted median survival times < 20 days for all cohorts, but all cohorts exhibited higher medial survival times. The cohorts injected with unsorted MSCs grown on 1 kPa PDMS and *D*^hi^ MSCs both exhibited median survival times of > 50 days. Additionally, the survival proportion for mice injected with MSCs grown on 1 kPa PDMS exhibited a much higher survival proportion (> 80%) than predicted at this experiment endpoint of 50 days. Both the unsorted MSCs grown on TCPS and 100 kPa PDMS exhibited lower median survival times of 39 days and 49 days, respectively, but were both higher than the PLSR model predicted.

Pairwise comparisons across survival curves showed that all treatments with MSCs yielded statistically significant (*p* < 0.05, calculated from Mantel-Cox or log-rank test) higher survival curves when compared to no-treatment (saline injection) controls, suggesting improved support of hematopoietic recovery. (Mice with no treatment exhibited a median survival time of 13 days, comparable to median survival times in previous studies of radiation rescue [30] but not predicted quantitatively by the PLSR model because there were, of course, no expression data corresponding to the no-treatment condition.) Moreover, mice injected with *D*^hi^ MSCs had a much higher hazard ratio (calculated from the Mantel-Haenszel method, for projecting difference in outcomes when survival curves are statistically indistinguishable) of 4.4 when compared to MSCs expanded on 1 kPa PDMS. That is, mouse survival was more than four times more likely for injection of mechanoprimed vs. sorted MSCs. When comparing MSCs grown on TCPS and 100 kPa PDMS, the cohort treated with MSCs grown on 100 kPa PDMS resulted in a higher median survival time, suggesting that MSCs mechanoprimed on 100 kPa PDMS still yielded an improvement in survival when compared to standard MSC expansion on TCPS. The weight recovery of the mice (see Additional file 1: Figure S3) was also indicative of animal health and recovery after induced hematopoietic failure.

**Fig. 5:**
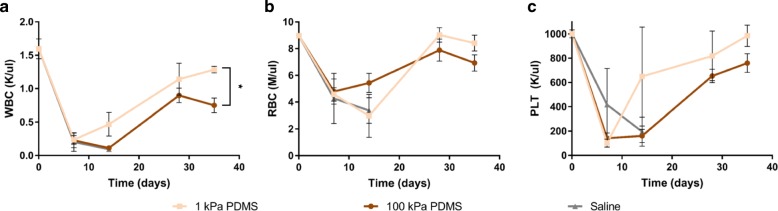
In vivo hematopoietic recovery: complete blood count (CBC). **a**-**c** CBC analysis was conducted at 7, 14, 28, and 35 days post-irradiation. Blood was collected from two experimental mouse groups: mice injected with MSCs grown on 1 kPa PDMS (*light orange*) and 100 kPa PDMS (*dark orange*). At early times of 7 and 14 days, the no-treatment saline control group (*gray*) was also included. Mean **a** white blood cell concentration, **b** red blood concentration, and **c** platelet concentrations are plotted with SEM. Blood was drawn from at least three mice per time point (*N* ≥ 3). Differences were calculated with one-tailed, Student’s *t* tests with unequal variance, (*p < 0.005)

In addition to animal weight and survival, we also analyzed the complete blood counts (CBCs) of mice from the no-treatment control group, and the two groups injected with unsorted MSCs grown on PDMS of lower and higher stiffness (Fig. 5). We drew blood from mice prior to the experiment and prior to irradiation (drawn multiple weeks before, but graphed at day 0) to determine the basal concentrations of white blood cells (WBCs), red blood cells (RBCs), and platelets (PLTs). At early time points of 1 week post-irradiation, we observed no differences in CBCs, as none of the mice had begun to recover. At 2 weeks post-irradiation, we observed that the WBC and PLT concentrations began to recover, indicated by upward trends, for mice injected with MSCs grown on the more compliant (1 kPa) PDMS (Fig. 5a, c). Interestingly, at this same time point, the RBC concentration began to recover for mice injected with MSCs grown on the stiffer (100 kPa) PDMS (Fig. 5b). At 4 weeks, mice injected with MSCs grown on PDMS of either stiffness recovered or exceeded the baseline minimum CBC for that cohort. At the last time point (5 weeks) at which we analyzed CBC, we observed that all blood and platelet counts were closest to basal levels for mice injected with MSCs grown on the more compliant PDMS.

## Discussion

Regression techniques such as linear Pearson correlations and PLSR are useful in identifying proteins that are correlated highly with improved survival or hematopoietic recovery (Table 1 and Fig. 1a-b). From both of these techniques, we identified eight proteins that were significantly correlated with mouse survival after irradiation injury indicative of improved hematopoietic recovery. While we do not claim those factors to be an exhaustive or even necessarily the most important list of MSC-secreted proteins that support radiation recovery, we found a subset of these to be positively and significantly correlated with in vivo outcomes. Thus, we deem these proteins or cytokines as those secreted factors that may play a direct role in supporting hematopoietic recovery, or at least convey strong correlations with that clinical endpoint. Moreover, using PLSR, we generated a predictive model (Fig. 1c-d) to predict survival trends of recovery from new sets of secretome expression data.

After creating a regression model to predict mouse survival, we applied the model to data obtained from a set of MSCs modulated with cell-material interactions. In a previous published study, we demonstrated that we could mechanically modulate the MSC secretome by growing the MSCs on PDMS substrata of varying stiffness [35]. Here, we used these same material mechanics to tune the MSC secretome for use as test data in our regression model of animal survival (Fig. 2a). To determine if these changes in expression could be physiologically relevant in supporting hematopoietic recovery, we then compared the expression of MSCs cultured on PDMS to that of *D*^hi^ MSCs and unsorted MSCs grown on TCPS and obtained from the same donor cell source (Fig. 2b). From our secretome characterization, we found that mechanically modulated MSCs can attain secretome expression levels similar to or higher than the *D*^hi^ cells (Fig. 2b) of the proteins correlated with survival (Table 1). Together, these data suggest that we can use material mechanics to modulate the unsorted MSCs secretome to approximate that of the therapeutically effective sorted MSCs, or *D*^hi^ phenotype. Importantly, under these conditions and at these time points, we have shown that the MSCs are not terminally differentiated as defined by metabolic expression [35].

We next validated in vitro that our mechanically modulated MSCs could have a direct effect on HSPC behavior. We observed an overall increase in HSPC proliferation when HSPCs were grown in co-culture with MSCs on our most compliant PDMS substrata. However, these trends in proliferation were highly variable and time dependent (Fig. 3a-b). For example, there were proportionally larger increases in HSPC proliferation at day 4 (~ 300–400% increase) than at day 7 (~ 50–100% increase). Additionally, for some replicate experiments, we noted the maximum increase in proliferation in the 10 kPa PDMS substrata instead of the more compliant 1 kPa substrata (Fig. 3a). We attributed this high variance to the fact that we used a different HSPC human donor for each independent experiment, with the potential for significantly different initial genetic profiles among HSPC donors. In the in vivo experiments, we were not subject to these same limitations, as we used MSCs obtained from a single donor source and administered to a single strain of mice.

In terms of surface marker expression, we observed higher CD123^+^ expression on HSPCs grown in co-culture with MSCs on the stiffest PDMS substrata (Fig. 3e). This suggests that at a co-culture time of 1 week, our MSCs grown on the stiffest PDMS substrata could prime the HSPCs towards myeloid lineages [45, 46]. Additionally, we observed a decrease in CD34^+^ expression concurrent with this increases in CD123^+^ (Fig. 3d). This suggests that while MSCs grown on the 100 kPa PDMS substrata may be useful for myeloid priming of the HSPCs, the MSCs grown on the 1 kPa PDMS may be better for maintaining the naivety of the HSPCs. Nevertheless, HSPCs grown with MSCs on 1 kPa PDMS expressed lower CD34^+^ than those grown on TCPS. These concurrent changes in CD123^+^ and CD34^+^ expression suggest that our mechanically modulated MSCs may play a direct role in regulating the proliferation and differentiation of HSPCs. In a separate study, we explored how non-contact co-culture affected HSPC behavior to ascertain whether or not these phenotypic changes were due to the MSC secretome or to direct cell-cell interactions [35]. We found that changes in proliferation were secretome mediated, but changes in surface marker expression required cell-cell contact. Other studies have suggested that HSPCs in monoculture may also be directly affected by substratum mechanics [47–51]. Nevertheless, our in vitro co-culture results demonstrate that mechanically modulated MSCs have the potential to regulate the differentiation and proliferation of the HSPCs. However, we do note that in this study we did not explore the functional phenotypes of the HSPCs after co-culture with our MSCs expanded on PDMS substrata, so this remains an area of future study.

We then explored how our mechanically modulated MSCs could affect hematopoietic recovery in vivo. We first used our PLSR model to predict the survival curves of the mice injected with MSCs grown on our mechanically distinct PDMS substrata. For these in vivo experiments, we chose to deliver and compare outcomes for MSCs grown on the stiffest (100 kPa) and most compliant (1 kPa) substrata, because those two cell populations yielded statistically significant differences in HSPC surface marker expression when grown in co-culture (Fig. 3d-e). Although our model predicted some relative trends in survival across experimental groups correctly, the predicted survival proportions were uniformly lower than the experimental data (Fig. 4a-b). We attribute this discrepancy in our PLSR model to the limited expression data used to construct the model. We used targeted, antibody-based techniques (Luminex and ELISAs) or targeted assays to characterize the concentration of only 35 proteins. However, there are likely other proteins that could play a role in supporting hematopoietic recovery, and these were not included in our regression model because the initial data set was constrained by a prudently targeted profiling approach. For example, we found that osteopontin is highly upregulated in our MSCs grown on PDMS substrata [35]. Osteopontin is also a marker for osteoprogenitors and of the *D*^hi^ MSC phenotype in our previous studies on hematopoietic recovery [30]. In the literature, osteopontin has been demonstrated to play a role in regulating the self-renewal capacity of HSPCs [44, 52, 53]. However, we were unable to incorporate osteopontin in our initial regression model as it was not identified at the time of those in vivo experiments to be correlative with survival outcomes, so we did not acquire osteopontin expression across all experimental groups used to construct our predictive model. A non-targeted approach to more wholly characterize the MSC secretome can identify additional proteins including osteopontin, which also may be correlative and crucial in supporting hematopoietic recovery; in that case, such additional data would then also improve the accuracy of the animal survival model.

In addition to observing mouse survival over the course of the experiment, we also tracked weight at multiple time points. Weight recovery was concurrent with improved survival, with surviving mice recovering approximately ≥90% of their original weight by day 35 (see Additional file 1: Figure S3 and Table S1). Mice with induced hematopoietic failure and then injected with MSCs grown on the most compliant (1 kPa) PDMS exhibited the highest survival proportion of 83.2% at the end of the experiment (Fig. 4b). This suggests that of all conditions tested, MSCs grown on compliant substrata of stiffness comparable to bone marrow [54, 55] were most efficient at supporting radiation rescue in vivo. Although we did not track human MSC biodistribution in the present study, our previous studies using the same animal and injury model have demonstrated that the MSCs home to, but do not engraft and are not detectable beyond day 10 in the bone marrow compartment [30]. Together, these findings demonstrate that by growing the unsorted MSCs on PDMS, therapeutic potential to support hematopoietic recovery is realized even more effectively than by sorting *D*^hi^ MSC subpopulations. The animal cohorts injected with *D*^hi^ MSCs and the unsorted MSCs on TCPS exhibited similar survival and weight recovery in the first 5 weeks of the experiment (see Fig. 4b and Additional file 1: Figure S3a-b).

Note that the *D*^hi^ MSCs in Fig. 4b (used in the present in vivo experiments used to validate the model) and *D*^hi^ MSCs in Fig. 1c (used in previously published in vivo experiments used to construct the model) were obtained from different human donors. Thus, the differing survival responses can also be attributed in part to this donor cell source variation, with corresponding biological variation in secretome profile. It is plausible that the therapeutic efficacy of the *D*^hi^ MSCs is donor dependent, and thus that biophysically sorting of donor cells maybe insufficient as a sole method to improve therapeutic efficacy in supporting hematopoietic recovery. However, our results demonstrate that mechanically modulated MSCs can robustly improve the cell secretory profile and improve radiation rescue, as compared to either sorted *D*^hi^ MSC subpopulations or unsorted MSCs grown on TCPS that were obtained from the same donor (Fig. 4b). In the present experiments, mechanoprimed MSCs resulted in a fourfold decrease in risk hazard (likeliness of survival, Fig. 4b) and also circumvented the fivefold reduction in cell yield by obviating the biophysical sorting of a subpopulation.

After mice nearly recovered initial weights at day 35, we obtained blood samples from multiple mice within cohorts that were injected with MSCs grown on 100 kPa or 1 kPa PDMS; we conducted cheek bleeds to obtain minimally sufficient blood volumes for CBC results that still facilitated continued recovery beyond day 35. Nevertheless, that procedure induced a minor injury to the recovering mouse (~ 10–20% blood volume loss) and resulted in subsequent weight loss. Mice injected with MSCs grown on 100 kPa PDMS continued to lose weight after this procedure, and did not recover initial weight at the end of the experiment (see Additional file 1: Figure S3 and Table S1). Interestingly, however, mice injected with MSCs expanded on the more compliant (1 kPa) PDMS ceased weight loss after day 42 (see Additional file 1: Figure S3A). At 49 days, or 2 weeks after re-injury, mice injected with MSCs expanded on 100 kPa PDMS exhibited significantly lower weight recovery (adjusted *p* < 0.05) than mice injected with MSCs expanded on 1 kPa PDMS (see Additional file 1: Figure S3 and Supplementary Table S2). This comparison suggests that mechanoprimed MSCs (expanded on 1 kPa PDMS) could support long-term hematopoiesis including recovery from re-injury after initial hematopoietic failure.

From the complete blood count (CBC) analysis (Fig. 5a-c), we observed that mice injected with MSCs grown on PDMS (1 and 100 kPa) recovered WBC, RBC, and PLT levels by day 35. At that time point, all three blood count components were highest for mice injected with the MSCs expanded on the more compliant PDMS (Fig. 5a-c). At day 35, WBC concentrations were significantly higher (*p* = 0.001, calculated from one-tailed Student’s *t* test with unequal variance) for cohorts receiving MSCs expanded on the more compliant PDMS compared with the stiffer PDMS substratum. However, RBC and PLT levels were insignificantly higher (*p* = 0.057 and *p* = 0.086, respectively) on the more compliant substratum, due to low sample size (*n* = 5 or 6 animals per cohort tested for CBC) and appreciable variance. Nevertheless, the recovery of all three blood components suggests that MSC populations that are mechanoprimed on compliant substrata (here, 1 kPa PDMS) can support more efficient recovery of the hematopoietic lineages. Note that our in vitro co-culture analysis suggested that mechanoprimed MSCs expanded on 100 kPa would promote myeloid progenitor priming of HSPCs. This in vitro prediction was also borne out by these in vivo experiments, at least at short recovery time points (<day 30). Specifically, at day 14 we observed a slightly higher concentration of RBCs (*p* = 0.13, *n* = 3) in the mouse cohort injected with MSCs grown on the stiffer (100 kPa) PDMS (Fig. 5b). Additionally, we observed a significant weight increase for that cohort (adjusted *p* values < 0.05, see Additional file 1: Table S2, with means for each cohort in Additional file 1: Table S1) at days 7 and 14, when compared with mice injected with *D*^hi^ MSCs; no other comparisons at those time points were statistically distinguishable. The hastened increase in RBC count in vivo suggests that this second type of mechanoprimed MSCs – expanded on stiffer PDMS than that required to enhance CBC recovery – could potentially support faster short-term recovery of RBCs and mouse weight than any other experimental condition. Thus, the in vitro co-culture may serve as a useful model to determine the short-term effects of how mechanoprimed MSCs can influence hematopoietic recovery.

## Conclusions

In this study, we mined previous in vitro and in vivo data to identify key factors in the MSC secretome that were correlated highly with improved radiation rescue in an in vivo mouse model of hematopoietic failure. Our regression model generally predicted survival post-irradiation accurately, including the improved survival outcomes for MSCs cultured on materials that were orders of magnitude more compliant that the typical polystyrene materials used for MSC expansion in preclinical studies. Despite these clear correlations between these MSC secretome elements and animal recovery, it remains an open question whether and how these identified factors play a role in directly supporting hematopoiesis. Nevertheless, we chose to explore how these identified secretome components could be used as predictive markers of an MSC phenotype that is therapeutically effective in promoting hematopoietic recovery.

We modulated the MSC secretome by tuning the mechanical properties of the cell culture substrata, engineering the cell culture-compatible material PDMS to range in stiffness from 10^3^ to 10^5^ Pa. We found that these mechanoprimed MSCs could modulate HSPC proliferation and differentiation in vitro (as indicated by surface marker expression) and support hematopoiesis in vivo. Most importantly, mechanoprimed MSCs resulted in markedly improved mouse survival, for a hematopoietic failure model induced by sublethal irradiation. This suggests that we could improve the MSC capacity to support hematopoietic recovery by simply culturing MSCs on compliant PDMS substrata that elicit the desired secretome profile.

The partial least squares regression (PLSR) model developed herein successfully predicted in vivo hematopoietic recovery from MSC secretion data. This approach should be applicable generally to prediction of the therapeutic efficacy of other MSC populations. This method of mechanopriming MSCs through modification of the material substratum stiffness is more scalable than previously reported sorting methods that isolated 20% of the cells as phenotypically desirable. In contrast, mechanopriming via the substratum mechanical cues affords that the unsorted, culture-expanded cell population will exhibit the predictably therapeutic phenotype. This validated modeling approach enables more efficient production of therapeutically viable MSCs for cell therapy applications in vivo*.*

## Additional file

Additional file 1: Supplementary Data and Figures. These supplementary data and figures include the variance explained in the PLSR model with varying numbers of model components. A more detailed three-dimensional rendering of Fig. 1a is rendered as a supplementary figure to depict specific secretome components included in training data. This supplementary section also includes the weight recovery of experimental cohorts during in vivo experiments along with the corresponding statistics. (ZIP 1863 kb)

## Abbreviations

ANG-1: Angiopoietin-1
BMP-2: Bone morphogenetic protein 2
CBC: Complete blood count
*D*^hi^ MSCs: Biophysically isolated mesenchymal stromal cells with large diameter (D > 20 μm)
EGF: Epidermal growth factor
ELISA: Enzyme-linked immunosorbent assay
FGF-1: Acidic fibroblast growth factor
HSC: Hematopoietic stem cell
HSPCs: Hematopoietic stem and progenitor cells
IGFPBP2: Insulin-like growth factor binding protein 2
IL-15: Interleukin-15
IL-6: Interleukin-6
IL-8: Interleukin-8
MCP-1: Monocyte chemoattractant protein-1
MSC: Mesenchymal stromal cell
OPN: Osteopontin
PDMS: Polydimethylsiloxane
PLSR: Partial least squares regression
PLT: Platelet
RANTES: Regulated on activation, normal T cell expressed and secreted
RBC: Red blood cell
SCF: Stem cell factor
SDF1: Stromal cell-derived factor-1
TCPS: Tissue culture polystyrene
THPO: Thrombopoietin
VEGF-A: Vascular endothelial growth factor A
WBC: White blood cell
