# Supplementary material for: Improving hematopoietic recovery through modeling and modulation of the mesenchymal stromal cell secretome
Source: Stem Cell Res Ther. 2018 Oct 24;9:268. doi: 10.1186/s13287-018-0982-2 (PMC6199758; doi:10.1186/s13287-018-0982-2)
Supplement: Supplementary file 1 — Supplementary Data and Figures. These supplementary data and figures include the variance explained in the PLSR model with varying numbers of model components. A more detailed three-dimensional rendering of Fig. 1a is rendered as a supplementary figure to depict specific secretome components included in training data. This supplementary section also includes the weight recovery of experimental cohorts during in vivo experiments along with the corresponding statistics. (ZIP 1863 kb) [file 13287_2018_982_MOESM1_ESM.zip › Additional File 1 - Final.docx]

**Supplementary Data and Figures**

**Improving hematopoietic recovery through modeling and modulation of the mesenchymal stromal cell secretome**, Liu et al.

1. **Variance Explained in PLSR Components**

To determine the number of principal components necessary to build our partial least squares regression (PLSR) space, we plotted the cumulative variance in both the predictor variable (X) and the response variable (Y) against the number of PLS components (Fig. S1). In Supplementary Fig. S1, over 90% of the variance is captured with a 2-component PLS model for both the predictor (Supplementary Fig. S1A) and the response (Supplementary Fig. S1B) variables, which represent the protein expression data and survival data from Poon et al. [1], respectively. The survival data acquired and implemented in the model was the survival proportion for each experimental cohort at every time point recorded during the 50-day experiment. Although a third component increases the variance explained in X and Y by 2.5% and 6.6%, respectively, these increases are small compared to the increases of the second component (26.4% and 44.6% for X and Y, respectively). Thus, to avoid overfitting the regression model with such limited model data, we chose to use a 2-component or 2-dimensional PLSR model.


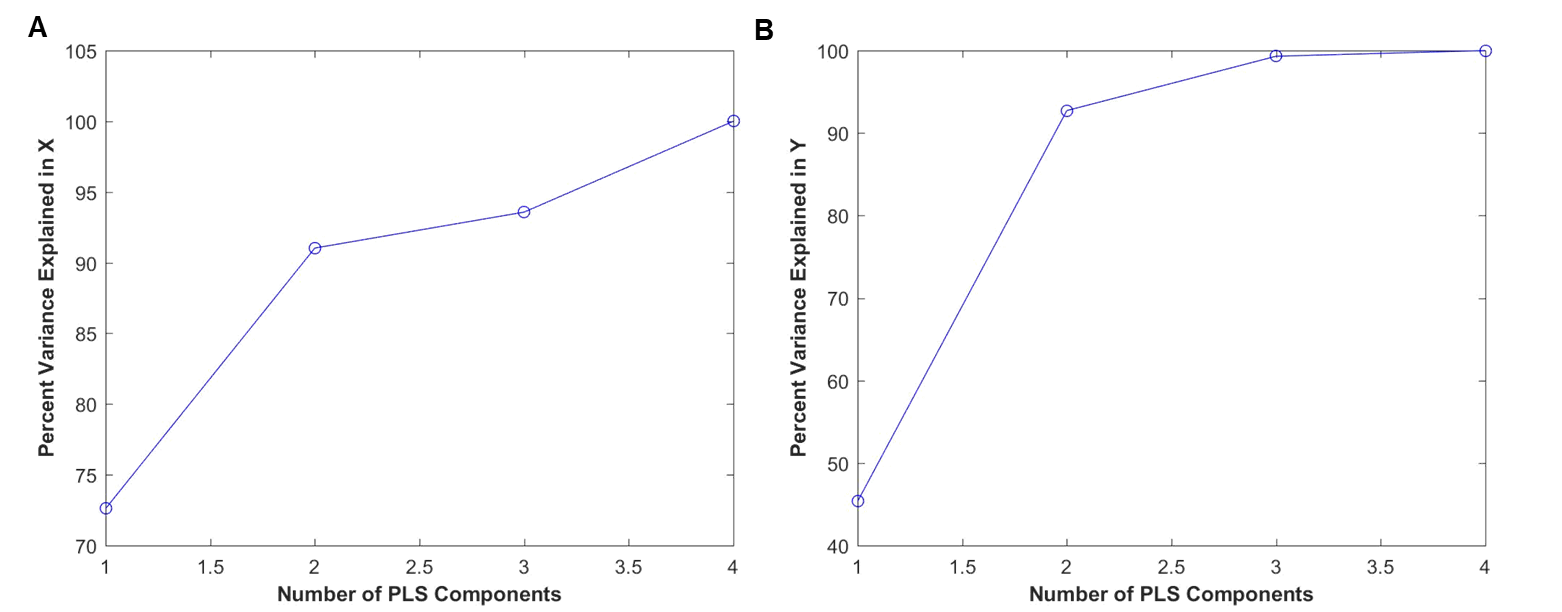
**Supplementary Figure S1. PLSR Components and Variance.** A) Percent variance explained in predictor variable, X, captured by 1 to 4 number of PLS components. B) Percent variance explained in response variable, Y, captured by 1 to 4 number of PLS components. Here, predictor data are protein expression and response data are mouse survival.

1. **Training Data in Detail**

For ease of visualization, we excluded secreted factor names and abbreviations from the training data set in Fig. 1A from the main manuscript. We have included the abbreviations of the secretome components (cytokines, chemokines, and growth factors) in the 3D plot of the training data below. To help visualize the factors that are correlative with survival, we have also color-coded the factor abbreviations along the axis that were statistically correlated with survival from either Pearson’s linear regression, partial least squares regression, or both. From this additional figure (Fig. S2), we could observe easily which set of peaks corresponded to which secreted factors.

**
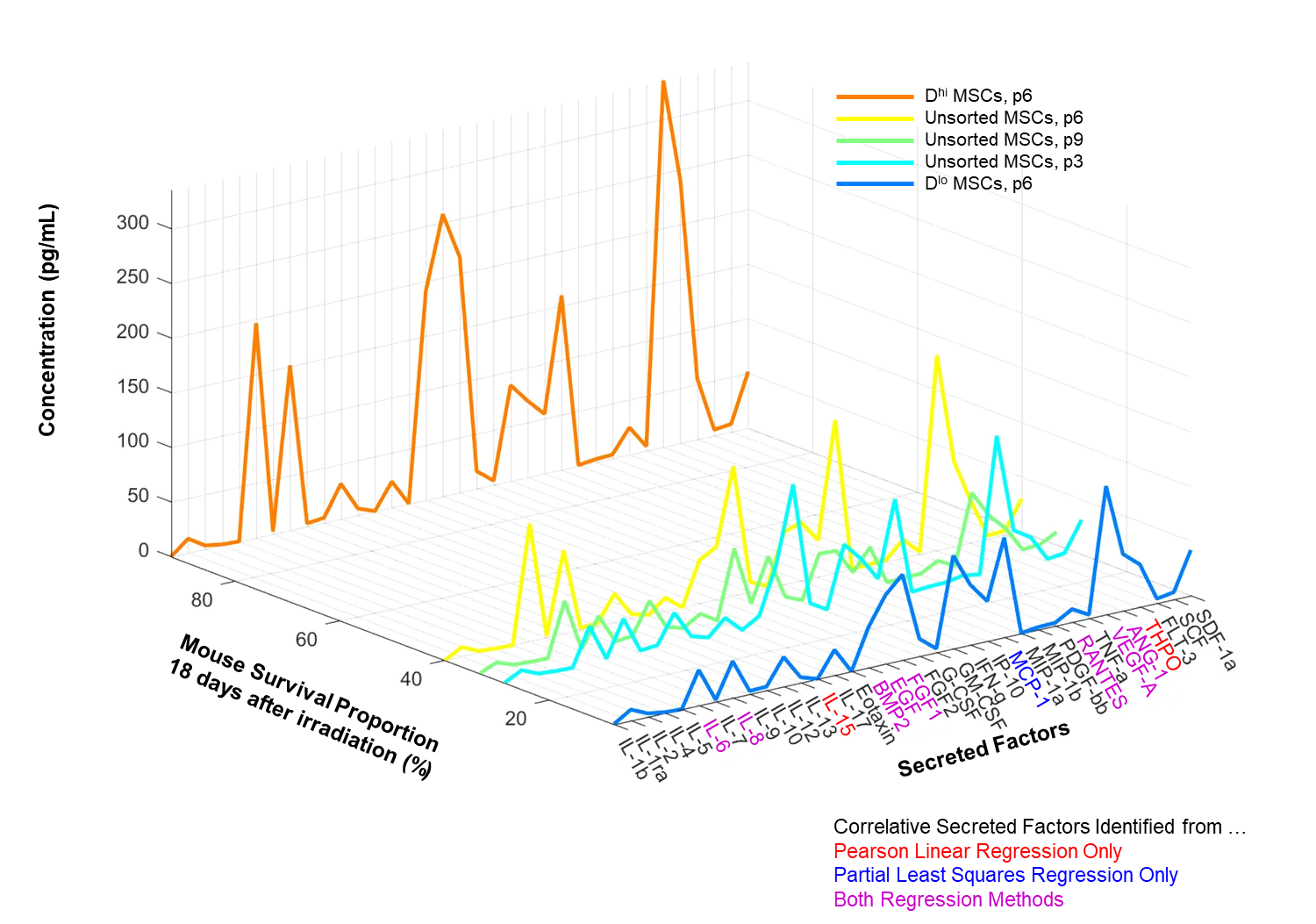
**

**Supplementary Figure S2. Secreted Factors versus Survival Proportion.** Re-creation of 3D plot of Figure 1A. All secreted factor abbreviations are listed to depict which factors correspond each set of expression data. Factors that only were identified to be correlative with survival from Pearson’s linear regression are written in red. Factors that only were identified to be correlative with survival from Partial Least Squares Regression are written in blue. Factors that were identified from both regression methods are written in purple.

1. **Weight Recovery**

Along with mouse survival (Fig. 4 in main manuscript), we also tracked the weight recovery of each mouse in all experimental groups (Fig. S3). Weight recovery was indicative of the overall health of the mice in each experimental group. Nevertheless, we could only track the weights of surviving mice, so differences in experimental groups are abrogated towards the end of the experiment when surviving mice are the ones that have recovered in each cohort. To more easily compare trends in weight recovery, we calculated the mean % weight recovery for all experimental cohorts at each week post-irradiation (Table S1). Although not statistically significant, we observed that the mice injected with unsorted MSCs grown on PDMS showed faster and greater weight recovery at around day 28 post-irradiation, when compared to those injected with the MSCs grown on TCPS. This higher mean weight recovery is recorded in Table S1 at day 28 post-irradiation.

We also observed a slight separation between the weight recovery curves of unsorted MSCs grown on PDMS (orange curves, Fig. S3A) and unsorted MSCs expanded on TCPS (Fig. S3B). The mean weight recovery for mice injected with unsorted MSCs expanded on TCPS failed to recover above 80% at the end of the experiment (Table S1). This suggests that mechanopriming unsorted MSCs by expanding them on PDMS can help support the weight recovery of the mice over standard culture conditions. For the *D*^hi^ MSCs expanded on TCPS, the weight recovery was more comparable to that of MSCs expanded on PDMS (Fig. S3C), but also appeared to decline in the last weeks of the experiment. Moreover, early on in the experiment (day 7 and day 14), mice injected with mechanoprimed MSCs grown on ~100 kPa PDMS appear to have the highest weight recovery that is statistically significantly higher than mice injected with *D*^hi^ MSCs (Fig. S3C and Table S2). This suggests that the mechanoprimed MSCs grown on ~100 kPa PDMS support faster, short-term recovery in the overall health of the mice than *D*^hi^ MSCs. Toward the end of the experiment (beyond day 40), we also observed a separation between cohorts injected with *D*^hi^ MSCs and MSCs mechanoprimed on ~1 kPa PDMS (Fig. S3C). However, the difference in weight recovery between these two groups was not statistically significantly different due to high variance. Moreover, as the experiment proceeded towards later times fewer mice survive, so we could not observe statistically significant differences across all experimental groups.

When comparing between the two groups injected with mechanoprimed MSCs (Fig. S3A, 100 kPa and 1 kPa substrata), we observed differences in mouse weight recovery towards the end of the experiment. After re-injury (day 35) due to cheek bleeding, the mice injected with mechanoprimed MSCs expanded on ~100 kPa PDMS declined in their weight and did not recover their original weight (~75% at day 49-50, Table S1). At the experiment conclusion (day 50), mice injected with MSCs expanded on ~1 kPa PDMS exhibited the highest overall weight recovery that was statistically significantly higher than MSCs expanded on ~100 kPa PDMS (Table S2). This suggests that the mechanoprimed MSCs expanded on ~1 kPa PDMS can better support recovery and maintenance of mice weight even after re-injury.


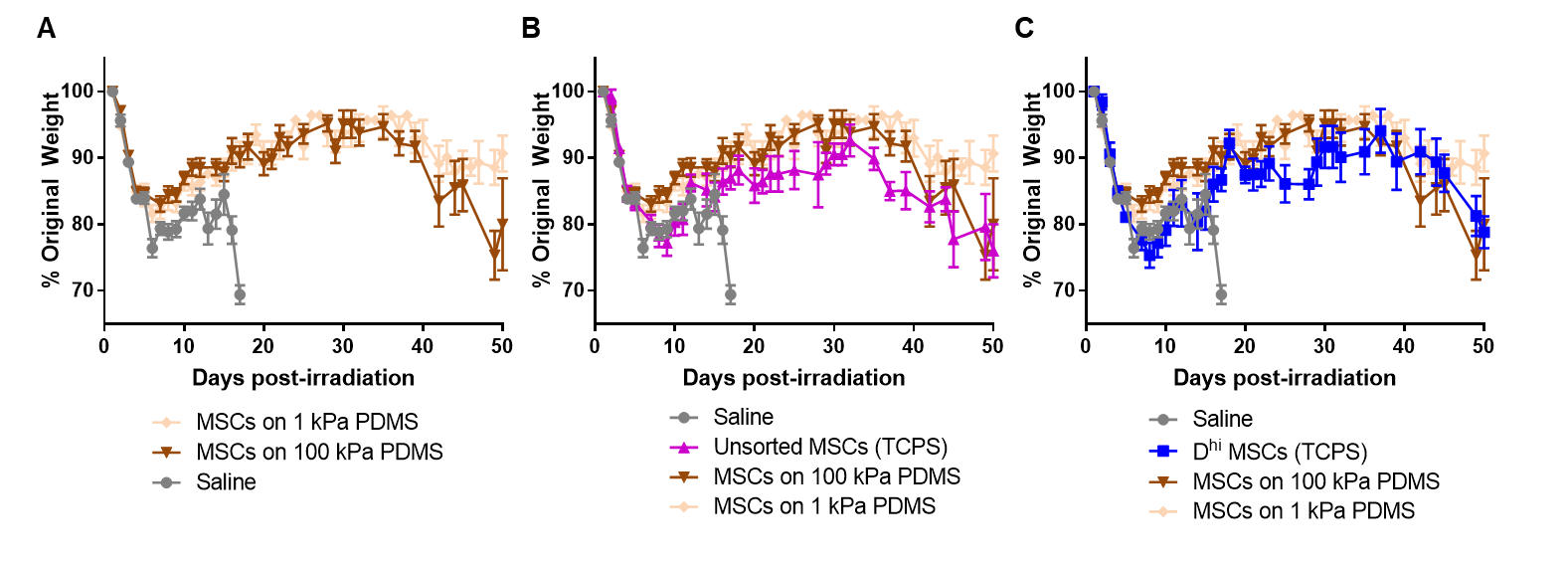
**Supplementary Figure S3. Weight Recovery of Sublethally Irradiated Mice**. Mouse models of hematopoietic failure were injected with MSCs from four experimental groups including unsorted MSCs grown on ~1 kPa PDMS (light orange), ~100 kPa (dark orange) PDMS, TCPS (purple) and D^hi^ MSCs grown on TCPS (blue). A no treatment or saline injection (cell-free) control is also included in gray. (A-C) Mice weights corresponding to each experimental group in Fig. 4 of the main manuscript were tracked over the course of 50-days after irradiation. Mean percentage of original weight values are plotted with ±SEM (N≥9 for all conditions). For easier visual comparison, mice weight recovery is plotted separately (A) for MSCs grown on PDMS conditions only, (B) all unsorted MSCs on PDMS and TCPS, and (C) sorted *D*^hi^ MSCs and MSCs expanded on PDMS.

**Supplementary Table S1. Mean Weight Recovery at Each Week.**

Mean weight recovery ± SEM was calculated each week post-irradiation with number of mice (n) included in parentheses. Note that numbers of mice in cohorts injected with MSCs expanded on PDMS (1 kPa and 100 kPa) and in the no-treatment control group were initially higher (n≥20) because mice were periodically culled for complete blood count anlaysis.

|  | **Mean % Original Weight** | | | | |
| --- | --- | --- | --- | --- | --- |
|  | **Saline** | ***D*^hi^ MSCs on  TCPS** | **Unsorted MSCs on**  **TCPS** | **Unsorted MSCs on  100 kPa PDMS** | **Unsorted MSCs on  1 kPa PDMS** |
| **Day 7** | 79.37 ±0.99 (n=24) | 76.83 ±1.43  (n=8) | 80.40  ±1.06  (n=11) | 83.07  ±1.17  (n=20) | 82.19  ±1.34  (n=25) |
| **Day 14** | 81.56  ±2.21  (n=18) | 80.42  ±4.28  (n=7) | 85.20  ±2.47  (n=8) | 88.61  ±1.21  (n=16) | 87.13  ±1.21  (n=19) |
| **Day 21** |  | 87.52  ±2.39  (n=5) | 86.46  ±1.79  (n=7) | 89.81  ±1.46  (n=13) | 90.75  ±1.77  (n=15) |
| **Day 28** |  | 86.05  ±2.28  (n=5) | 87.48  ±4.91  (n=7) | 95.21  ±1.19  (n=13) | 93.72  ±1.92  (n=15) |
| **Day 35** |  | 90.93  ±3.40  (n=5) | 89.89  ±1.66  (n=6) | 94.72  ±1.90  (n=10) | 95.44  ±2.35  (n=14) |
| **Day 42** |  | 90.93  ±3.40  (n=5) | 82.65  ±2.34  (n=5) | 83.47  ±3.83  (n=9) | 89.14  ±2.70  (n=14) |
| **Day 49** |  | 81.29  ±3.02  (n=5) | 79.61  ±4.96  (n=3) | 75.36  ±3.66  (n=5) | 88.57  ±2.64  (n=12) |

**Supplementary Table S2. Statistical Comparisons Across Experimental Groups**

|  | **Tukey's multiple comparisons test** | | | | | | |
| --- | --- | --- | --- | --- | --- | --- | --- |
|  | **Adjusted p value** | | | | | | |
|  | **Day 7** | **Day 14** | **Day 21** | **Day 28** | **Day 35** | **Day 42** | **Day 49** |
| ***D*^hi^ MSCs vs. Unsorted** | 0.5132 | 0.5091 | 0.9896 | 0.9887 | 0.9952 | 0.5377 | 0.9928 |
| ***D*^hi^ MSCs vs. 100 kPa** | 0.0439 | 0.043 | 0.8801 | 0.1245 | 0.778 | 0.5200 | 0.691 |
| ***D*^hi^ MSCs vs. 1 kPa** | 0.0917 | 0.1145 | 0.7143 | 0.2315 | 0.6381 | 0.9843 | 0.3951 |
| **Unsorted vs. 100 kPa** | 0.5738 | 0.6368 | 0.6202 | 0.1569 | 0.578 | 0.9987 | 0.9015 |
| **Unsorted vs. 1 kPa** | 0.8078 | 0.8992 | 0.3949 | 0.3003 | 0.4129 | 0.5780 | 0.3823 |
| **100 kPa vs. 1 kPa** | 0.9505 | 0.9128 | 0.9748 | 0.9555 | 0.9951 | 0.5268 | 0.0378 |

Entries highlighted in green are statistically significant, with adjusted p-values < 0.05. P-values were adjusted to account for family-wise error due to the multiple pair-wise comparisons made across experimental groups at every time point.
