# Supplementary material for: Improving hematopoietic recovery through modeling and modulation of the mesenchymal stromal cell secretome
Source: Stem Cell Res Ther. 2018 Oct 24;9:268. doi: 10.1186/s13287-018-0982-2 (PMC6199758; doi:10.1186/s13287-018-0982-2)
Supplement: Supplementary file 1 — Supplementary Data and Figures. These supplementary data and figures include the variance explained in the PLSR model with varying numbers of model components. A more detailed three-dimensional rendering of Fig. 1a is rendered as a supplementary figure to depict specific secretome components included in training data. This supplementary section also includes the weight recovery of experimental cohorts during in vivo experiments along with the corresponding statistics. (ZIP 1863 kb) [file FigureS2.pdf › FigureS2.pdf]

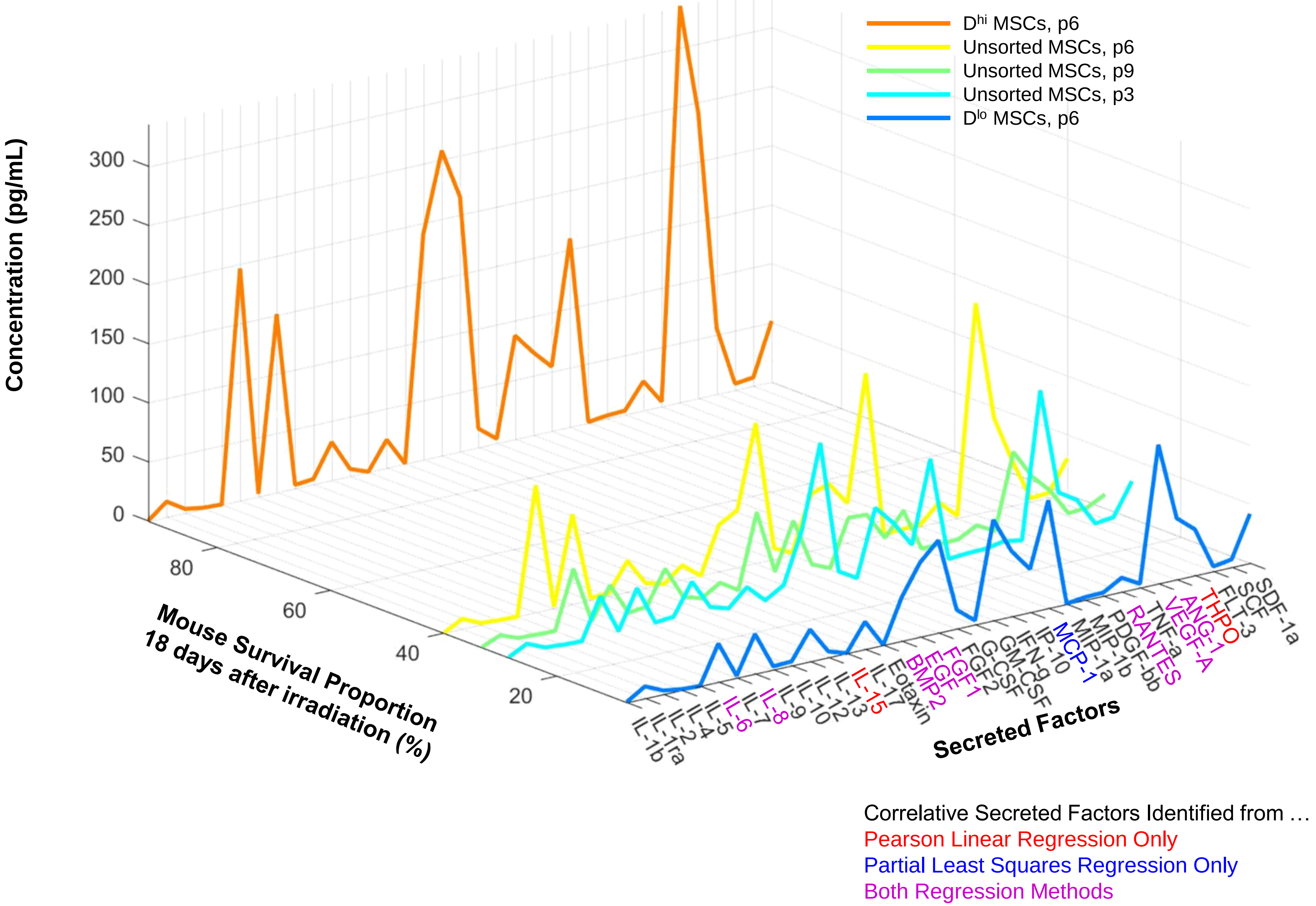
Concentration (pg/mL)
300
250
200
150
100
50
0
Mouse Survival Proportion
18 days after irradiation (%)
80
60
40
20
Secreted Factors
IL-1b
IL-1ra
IL-2
IL-4
IL-5
IL-6
IL-7
IL-8
IL-9
IL-10
IL-12
IL-13
IL-15
IL-17
Eotaxin
BMP2
EGF
FGF1
FGF2
G-CSF
GM-CSF
IFN-g
IP-10
MCP-1
MIP-1a
MIP-1b
PDGF-bb
RANTES
TNF-a
VEGF-A
ANG-1
THPO
FLT-3
SCF
SDF-1a
D^hi MSCs, p6
Unsorted MSCs, p6
Unsorted MSCs, p9
Unsorted MSCs, p3
D^lo MSCs, p6
Correlative Secreted Factors Identified from …
Pearson Linear Regression Only
Partial Least Squares Regression Only
Both Regression Methods
